# Synthesis of nano- alumina powder from impure kaolin and its application for arsenite removal from aqueous solutions

**DOI:** 10.1186/2052-336X-11-19

**Published:** 2013-07-16

**Authors:** Ahmad Khodadadi Darban, Yaser Kianinia, Ehsan Taheri-Nassaj

**Affiliations:** 1 Department of Environmental Engineering, Tarbiat Modares Environmental Research Center, Tarbiat Modares University, Tehran, Iran; 2 Department of Mineral Processing Engineering, Faculty of Engineering, Tarbiat Modares University, Tehran, Iran; 3 Department of Materials Engineering, Faculty of Engineering, Tarbiat Modares University, Tehran, Iran

**Keywords:** Nano- alumina, Arsenite, Removal, Isotherm, Kaolin

## Abstract

Adsorption is considered a cost-effective procedure, safer to handle with high removal efficiency. Activated alumina is the most commonly used adsorbent for the removal of arsenic from aqueous solutions. However, activated alumina has a low adsorption capacity and acts kinetically in a slow manner. An ideal adsorbent should have a high surface area, physical and/or chemical stability and be inexpensive. To meet this requirement, nanomeso porous γ-alumina with a high surface area (201.53 m^2^/g) and small particle size (22–36 nm) was prepared from inexpensive kaolin as the raw material, by precipitation method. The research results showed that adsorbent has the high adsorption capacity (for initial arsenite concentration up to 10 mg/L, in which 97.65% recovery was achieved). Optimal experimental conditions including pH, initial arsenite concentration and contact time were determined. Langmuir, Freundlich and Dubinin– Radushkevich isotherm models were applied to analyze the experimental data. The best interpretation for the experimental data was given by Langmuir adsorption isotherm equation and the maximum arsenite adsorbed by synthesized nano γ–alumina (qe) was found to be 40 (mg/g).

## Introduction

Arsenic is one of the most toxic known contaminants and has been recognized as a toxic element for centuries. Arsenic contamination has brought about severe health problems, such as lung, skin, liver and kidney cancers [1]. The chronic poisoning symptoms are hair loss, weight loss, chronic fatigue and diabetes [1-4]. A Long- period poisoning, leads to chronic disorders in many devices, from the nervous and cardio-vascular systems. Arsenite is found primarily as H_3_AsO_3_, H_2_AsO^3−^, HAsO_3_^2−^, and AsO_3_^3−,^ is more toxic than arsenate and is metabolized faster and easily accumulated in nails and hair [1-4].

The World Health Organization (WHO) sets a maximum allowable value of 10 mg/L for the arsenic concentration in drinking water [5].

There are several methods available for removal of arsenic from water and wastewater. The most effective treatments are reverse osmosis [6], ion exchange [7] and the adsorption on granular ferric hydroxide [8]. Adsorbent materials like activated alumina (AA) [9] anhydrous nanostructure iron (III)–titanium (IV) binary mixed oxide has been used for As(III) and As(V) removal [10].

Coagulation has also been used for Arsenic removal. The most commonly used metal salts are ferric salts such as ferric chloride or ferric sulfate and aluminum salts such as alum [11,12]. But these treatment systems are very expensive and some of them may produce large amounts of chemical sludge which before being disposed requires further treatment [13] and are not suitable.

Because of the high removal efficiency, convenience and simplicity, the adsorption process is disputably one of the more popular methods for the removal of arsenic ions. Among the adsorbents, nano γ–alumina with a high surface area has a high adsorption capacity and has been used for reducing different contaminants including nitrate [14] and As(V) [15] in water. Also, nano γ–alumina modified with 2,4-dinitrophenylhydrazine has been used for the removal of heavy-metal ions like Pb(II), Cd(II), Cr(III), Co(II), Ni(II) and Mn(II) in wastewater samples [16].

For the removal of arsenite, Most of these technologies are not efficient enough; hence, they are mainly applied for the removal of As (V). Therefore, a pre-oxidation step is usually required to transform the arsenite to As (V). The oxidation procedure is mainly performed by the addition of chemical reagents, such as potassium permanganate, chlorine dioxide, monochloramine, hypochlorite, ozone, hydrogen peroxide, or manganese oxide [17,18].

The objective of this article is offered in two sections, in the first section, a simple method for synthesizing of nano γ–alumina from impure kaolin is proposed, X-ray diffraction (XRD), scanning electron micrographs (SEM), Fourier Transform Infrared Spectroscopy (FTIR) and Brunauer Emmett Teller (BET) have been conducted for characterization of synthesizednano γ–alumina; and in the second section, the application of synthesized nano γ–alumina for arsenite removal is discussed. Adsorption studies were conducted under various experimental conditions, such as pH, contact time and initial arsenite concentrations. The data from the experiments were fitted with different models to identify the adsorption mechanism.

## Experimental

### Materials

All chemicals used are analytical reagents. The kaolin used came from Hamedan province, west of Iran. The arsenite stock solution was prepared using reagent-grade As_2_O_3_ (Merck).

### Synthesis of nano γ–alumina

At the first step, the kaolin was purified. Description of the purification of the kaolin is given elsewhere [19]. The kaolin was calcined into metakaolin at a heating rate of 10°C/min and the sample was kept for 2.5 h at 800°C to loosen the alumina components. Chemical composition of Kaolin and Metakaolin is shown in Table 1. Then, the metakaolin was leached with concentrated hydrochloric acid (6 M) at 90°C under stirring for 3.5 h, then, suspension was filtered and the filtrate was collected for producing of aluminum hydroxide. After that, following addition of polyethylene glycol (PEG, molar mass 4500), ammonia (0.75 M) was added. The precipitated Al (hydr) oxide was filtrated, washed with de-ionized water and dried. Finally, Subsequent calcination at 700°C yielded nano-sized γ-Al_2_O_3_[20]. Table 2 shows the composition of synthesised Nano γ-Al_2_O_3_.

**Table 1:** Chemical composition of Kaolin and Metakaolin (calcined at 700°C)

**Substance**	**Purified Kaolin**	**Metakaolin**
Na_2_O	0.117	0.131
MgO	0.113	0.119
Al_2_O_3_	35.366	44.958
SiO_2_	46.196	50.598
P_2_O_5_	0.166	0.148
SO_3_	0.078	0.051
TiO_2_	0.471	0.527
Fe_2_O_3_	0.386	0.371
CaO	0.058	0.055
LOI٭	17.01	3.04

٭Loss on ignition.

**Table 2:** **Chemical composition of synthesized nano γ-Al**_**2**_**O**_**3**_

**Substance**	**Nano γ-Al**_**2**_**O**_**3**_
MgO	0.11
Al_2_O_3_	90.021
SiO_2_	0.376
P_2_O_5_	0.188
Cl	0.233
SO_3_	0.027
Zn	0.24
Fe_2_O_3_	0.648
CaO	0.087
Pb	0.029
Ga	0.011
LOI	8.03

### Characterization of the adsorbent

A BET nitrogen absorption for the surface areas of the samples and scanning electron microscopy (SEM) were used to determine the particle size and morphology. Sample phases were identified using X-ray diffractometry (XRD) with nickel-filtered Cu K*α* radiation [21].

### FTIR analysis

To determine arsenite sorption mechanisms, two representative samples of nano γ–alumina before and after the arsenic sorption were subjected to FTIR, and compared [22]. The samples were first ground and then mixed with KBr at a sample-to- KBr ratio of 5/95 by weight. Then, after pressing the mixtures at 10 metric tons for a minute, the specimens were then scanned and characterized using an IR 100 spectrometer (Nicole) over the wave number ranging from 400 to 4000 cm^-1^.

### Measurement of the pH_pzc_ of nano γ–alumina by titration

pH_pzc_ (pH value at the point of zero charge) has important effects on adsorption capacity and was estimated by mass titration method [23]. Synthesized Nano γ–alumina suspensions, with the same solid contents were introduced in glass. The bottles were filled with nano γ–alumina. Then, the bottles were kept in a glove box at nitrogen atmosphere yet in the air, suspension stirring was intermittent but the bottleswere maintained at a constant temperature of 25°C. To reach pH equilibrium, the pH of the suspensions was measured after 24 h of contact time. Suspension pH was plotted versus the logarithm of the mass content. The point of zero charge value of the synthesized nano γ–alumina was considered the pH value of suspension which had the higher solid content when the pH evolution with solid concentration was low.

### BET analysis

N_2_ adsorption/desorption experiments for synthesisedNano γ-Al_2_O_3_ were carried out using a Belsorp mini II (BelJapan), and pore size distributions were calculated using the Barret-Joyner-Halenda (BJH) model on the desorption branch [20].

### Arsenic analysis

Arsenite in the solution was analyzed using UV – vis spectrophotometer (Cecil-model-7600) (detection limit: 3.4 μg/L with accuracy < ±5%) using the method described by Afkhami et al. [24].

The arsenite in samples were analyzed, based on their inhibition effect on the redox reaction between bromate and hydrochloric acid. In the spectrophotometric method, the decolorization of methyl orange with reaction products was used to monitor the reaction spectrophotometrically at 525 nm. The absorbance value was compared with a standard calibration curve.

### Arsenic adsorption studies

Approximately 0.02 g of synthesized nano γ–alumina was added to a capped tube containing 20 milliliters of a solution prepared at a predetermined arsenite concentration using deionized water, followed by shaking at room temperature for 3 h. The solutions were stirred continuously at a constant temperature to achieve equilibrium. After equilibrium, the solid and liquid were separated using a centrifuge (6000 rpm for 6 min). The aqueous phases were analyzed for arsenite content by spectrophotometer within 24 hours. Reproducibility of the measurements was determined in triplicates and the average values were reported. Relative standard deviations were discovered to be within ±4.0%.

The amount of arsenite adsorbed (q_e_(mg/g)) was calculated as follows:

(1) $${\mathrm{qe}=((C}_{0}\;{‒\;C}_{e})\times v)/m$$

In this equation, C_0_ and C_e_ are the initial and equilibrium concentrations of arsenite in solution (mg/L), v is the volume of solution (L) and m is mass of the adsorbent (g).

### pH studies

To determine the optimum pH for the maximum removal of arsenite, the equilibrium adsorption of arsenite with an initial concentration of 20 mg/L was investigated over a pH range of 3.5–8.5 because synthesizednano γ-Al_2_O_3_ was insoluble and stable within the this range of pH.Also,when the initial concentration was low, the arsenite concentration remaining in the solution after adsorption was below the detection limit of spectrophotometer, therefore, initial concentration of 5 mg/L was selected.

The initial pH of the solution was adjusted by using 0.1 M NaOH or 0.1 M HCl. 0.02 g synthesized nano γ–alumina was added to 20 mL solution. The mixture was shaken using a temperature-controlled water bath shaker at room temperature. After adsorption, the equilibrium arsenite of all solutions was measured and the value providing the maximum arsenite removal was determined.

### Kinetic studies

For achieving the rate of adsorption of arsenite, experiments were done at different time intervals (5 min–6 h). The minimum contact time was selected 5 minutes, because before that time the adsorption capacity was low. At upper than 6 h, trend of the plots showed that arsenite uptake had slower removal that gradually reached a plateau. In kinetic studies, 20 mL arsenite solution (20 mg/L) was adjusted to have a pH of 7.5 ± 0.1 by adding 0.1 M HCl and/or NaOH, and was agitated with synthesized nano γ–alumina (0.02 g) using a temperature-controlled water bath shaker at room temperature. After a fixed time interval, the adsorbent was separated and the aqueous phase was analyzed for determining the equilibrium concentration of arsenite. For reaching the adsorption equilibrium, experiments were repeated for different periods.

### Equilibriumadsorption studies

The adsorption of arsenite on synthesized nano γ–alumina was done at room temperature (25 ±1°C) by batch experiments. 20 milliliters of arsenite solution of varying initial concentrations (20–250 mg/L) with an initial solution pH of 7.5 in 20 mL capped tubes were shaken with 0.02 g of synthesized nano γ–alumina after adjusting the pH to the desired value, for a designated period of contact time in a temperature-controlled shaking assembly. After equilibrium, samples were centrifuged and the aqueous phase was then analyzed for residualarsenite concentration by spectrophotometer.

## Results and discussion

**Figure 1:**
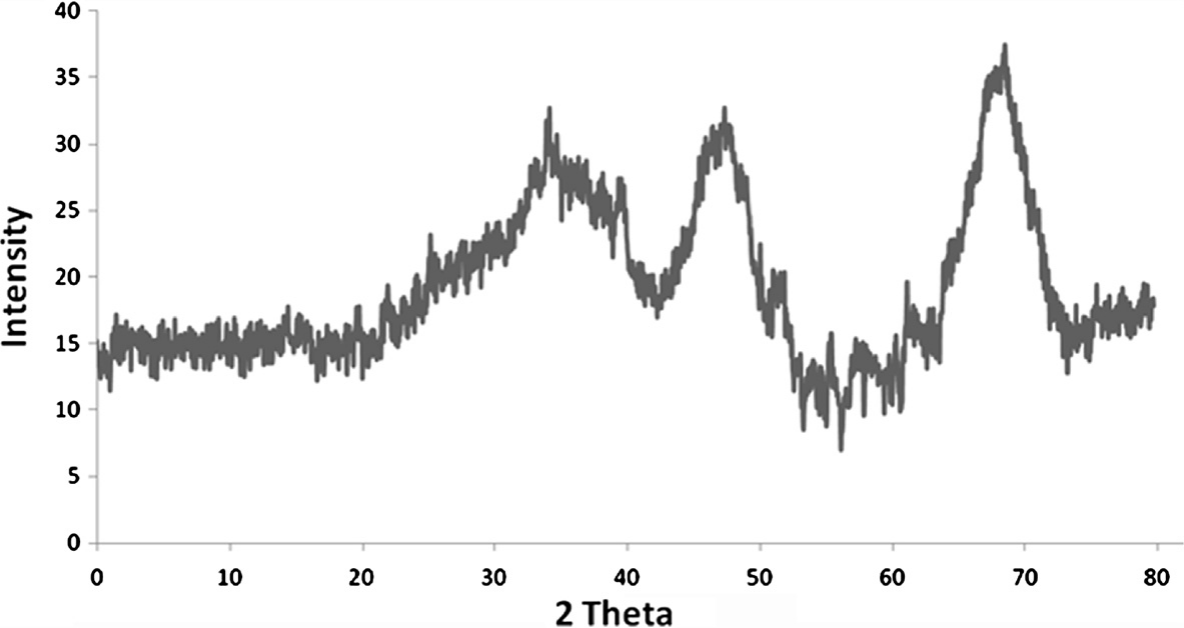
XRD pattern of the synthesized nano γ–alumina.

**Figure 2:**
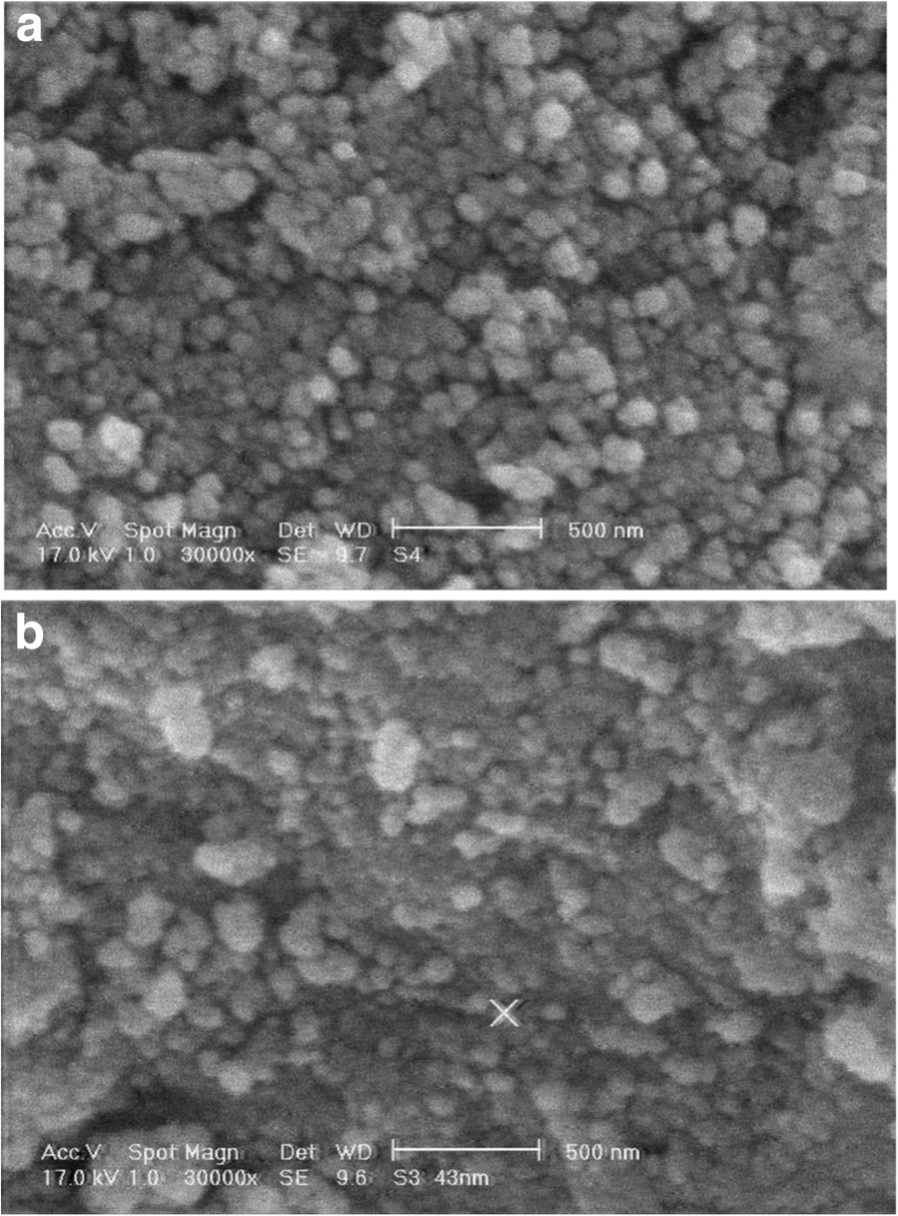
SEM graph of synthesized nano γ–alumina before (a) and after (b) adsorption of arsenite.

Figure 1 shows the XRD pattern of synthesized nano γ–alumina powder, the three main reflections of nano γ-Al_2_O_3_ phase are obviously observed as broad peaks at 2*θ* angles around 38.0◦, 46.0◦, and 66.0◦ which correspond to the (3 1 1), (4 0 0), and (4 4 0) planes, respectively. The peaks in the pattern are significantly indicated the formation of nano sized γ-Al_2_O_3_ crystallites. Figure 2 shows the SEM micrograph for γ-Al_2_O_3_ powders before and after adsorption of arsenite. The γ-Al_2_O_3_ before adsorption of arsenite (Figure 2a) indicated low agglomeration of particles with uniform sizes and spherical in shape. After adsorption of arsenite (Figure 2b) high agglomeration and non-uniform size of nano γ–alumina was seen.

**Figure 3:**
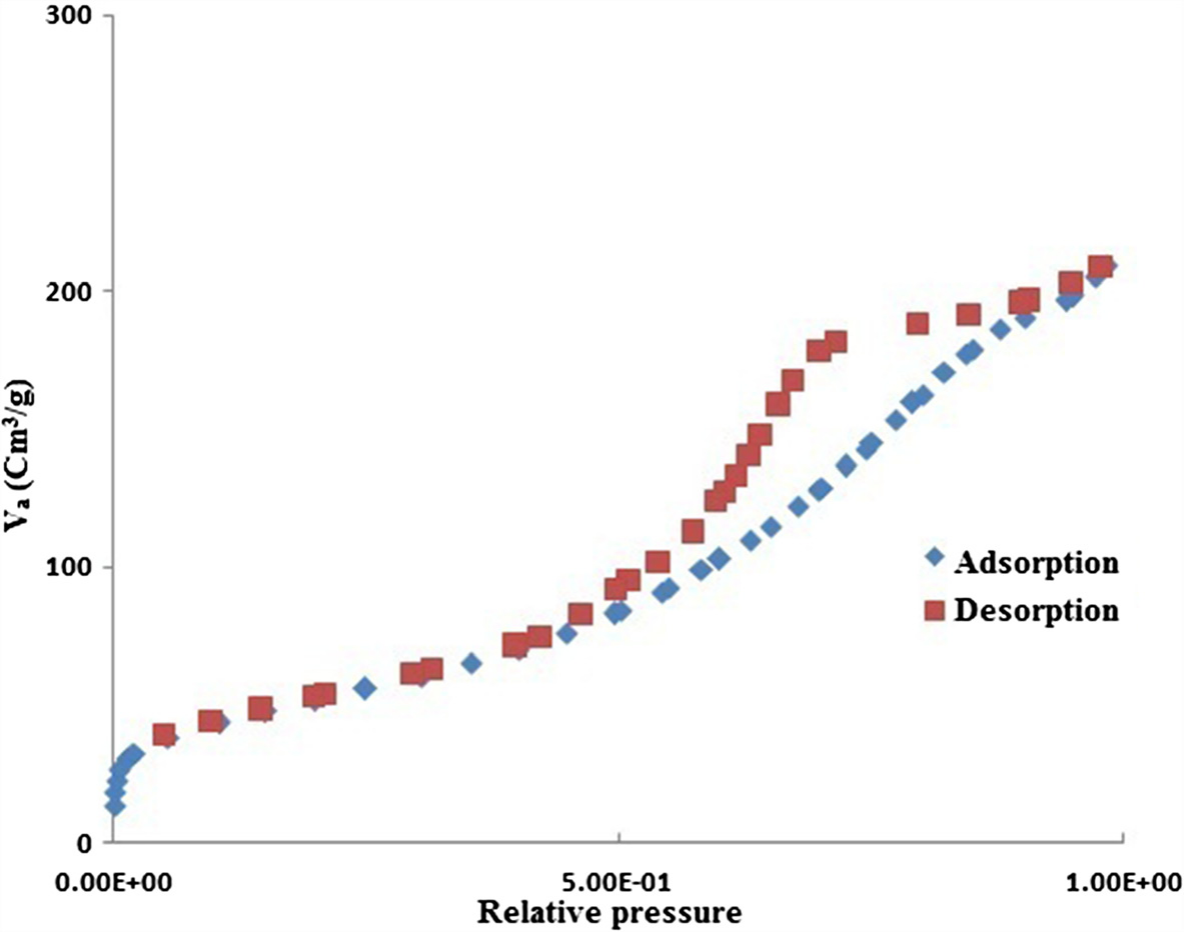
N_2_ adsorption/desorption isotherm for synthesisedNano γ - Al_2_O_3_.

Synthesisednano γ-Al_2_O_3_ showed a narrow pore size distribution with a high surface area (201.53 m^2^/g), a mean pore diameter of 6.91 nm and a high pore volume (0.33 cm^2^/g). The location of the hysteresis loop in the N_2_ isotherm obtained nano γ-Al_2_O_3_ sample (Figure 3: adsorbed volume of N_2_per gram(v_a_) against relative pressure) displays type IV hysteresis showing the presence of mesoporosity that can be used to determine whether the material possessed a regular framework pore or interparticle voids, such as a textural pore. The framework porosity at 0.4- 0.75 P/Po (relative pressure) in the N_2_ isotherm shows that the porosity was framework, while the textural porosity at 0.8-1 P/Po indicates porosity arising from the noncrystalline intra-aggregate voids and spaces formed by interparticle contacts.

**Figure 4:**
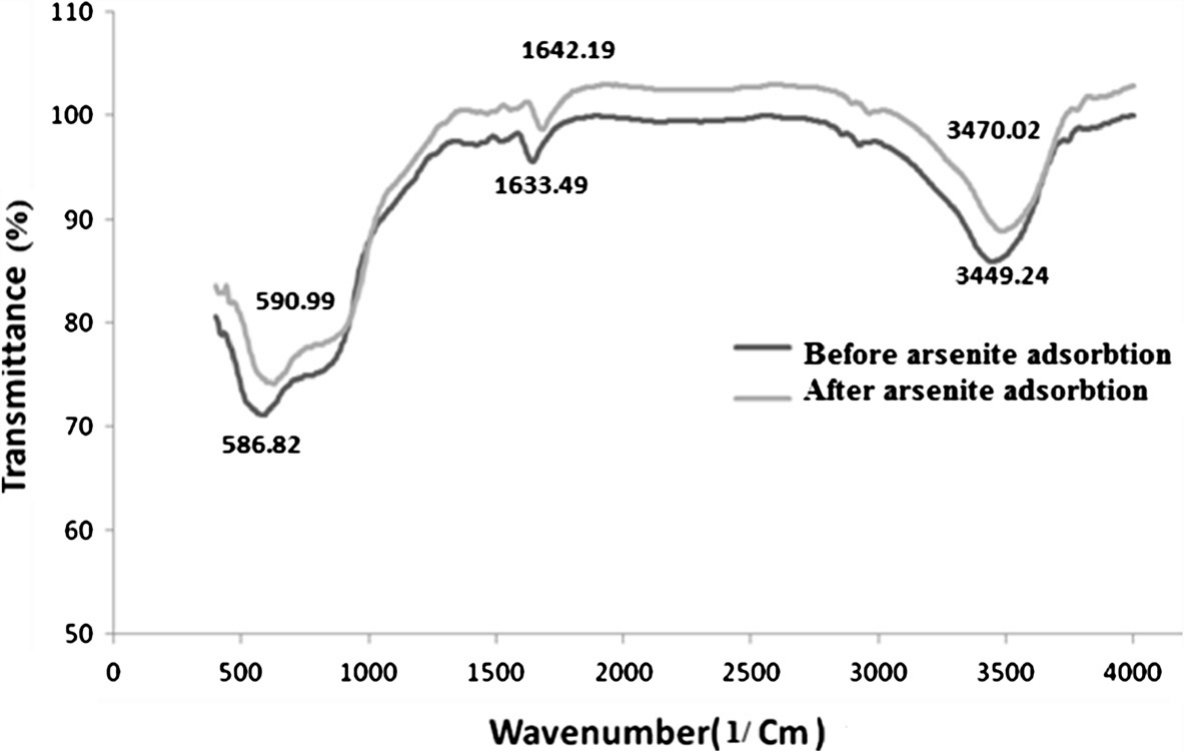
FT- IR spectra of the synthesized nano γ-alumina before and after arsenite adsorption.

According to FTIR spectra (Figure 4) the bands in the region of 400–1000 1/cm are generally associated with the stretching vibration of Al–O bonds [22] and broad bands around 3500 1/cm and 1630 1/cm are assigned to stretching and bending modes of adsorbed water. The bands at 500–750 1/cm are assigned to ν-AlO_6_, whereas the band around 900 1/cm corresponded to ν-AlO_4_[25].

The bands from 1400 1/cm to 1600 1/cm indicate the formation of alumina [26]. After adsorption of arsenite no peaks were seen and both FT- IR spectra were the same.

### Effect of pH on arsenite removal

**Figure 5:**
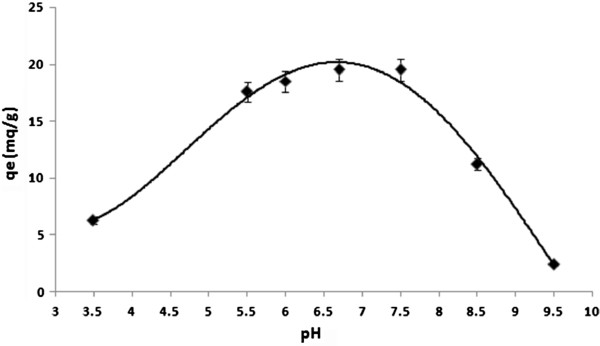
Effects of pH on arsenite adsorption (temperature: 25°C, initial arsenite concentration: 20 mg/L, contact time: 3 h, adsorbent dose: 1 g/L).

At the water-adsorbent interfaces, the pH is an important parameter controlling the sorption process. Figure 5 shows that the adsorption of arsenite on synthesizednano γ–alumina is strongly pH dependent.

High adsorption was observed in the pH range of 5.5–7.5 and low adsorption was achieved at higher pH values (higher than 8). In this pH range the non-ionic H_3_AsO_3_ is the dominant species, and van der waal force between the solute and the synthesized nano γ–alumina surface is expected. A further experiment was conducted to get the optimum pH in range of 5.5–7.5 and at pH=7.5 the maximum percent removal (97.65%) was achieved.

**Figure 6:**
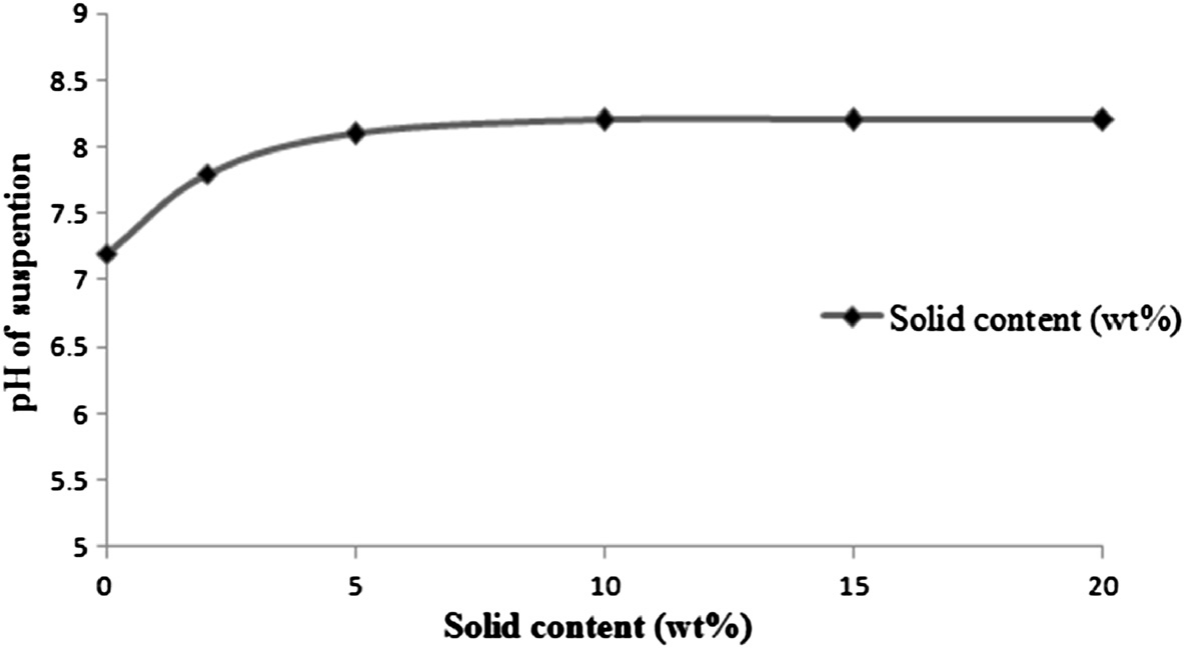
Determination of the point of zero charge of synthesized nano γ–alumina.

pHvalue of H_3_AsO_3_ is 9.2 indicating arsenite exists as a neutral specie up to a pH of about 9.2 [27] and at higher pH values it has a negative charge and pH_pzc_ of synthesized nano γ–alumina is 8.2 (Figure 6) signifying at pH >8.2, synthesized nano γ–alumina surface is negatively charged and at pH <8.2, synthesized nano γ–alumina surface is positively charged.

The reason for decreasing arsenite adsorption at 7.5< pH< 8.2 may be due to the competition for the active sites by OH^−^ ions and arsenite species. Based on the above result, the operating pH for the subsequent experiments was selected as 7.5±0.1.

### Effect of initial arsenite concentration and contact time on arsenite removal efficiency

The adsorption of arsenite on synthesized nano γ–alumina was investigated as a function of contact time (5 min– 6 h) at two different initial arsenite concentrations of 10 and 20 mg/L with an initial solution pH of 7.5.

**Figure 7:**
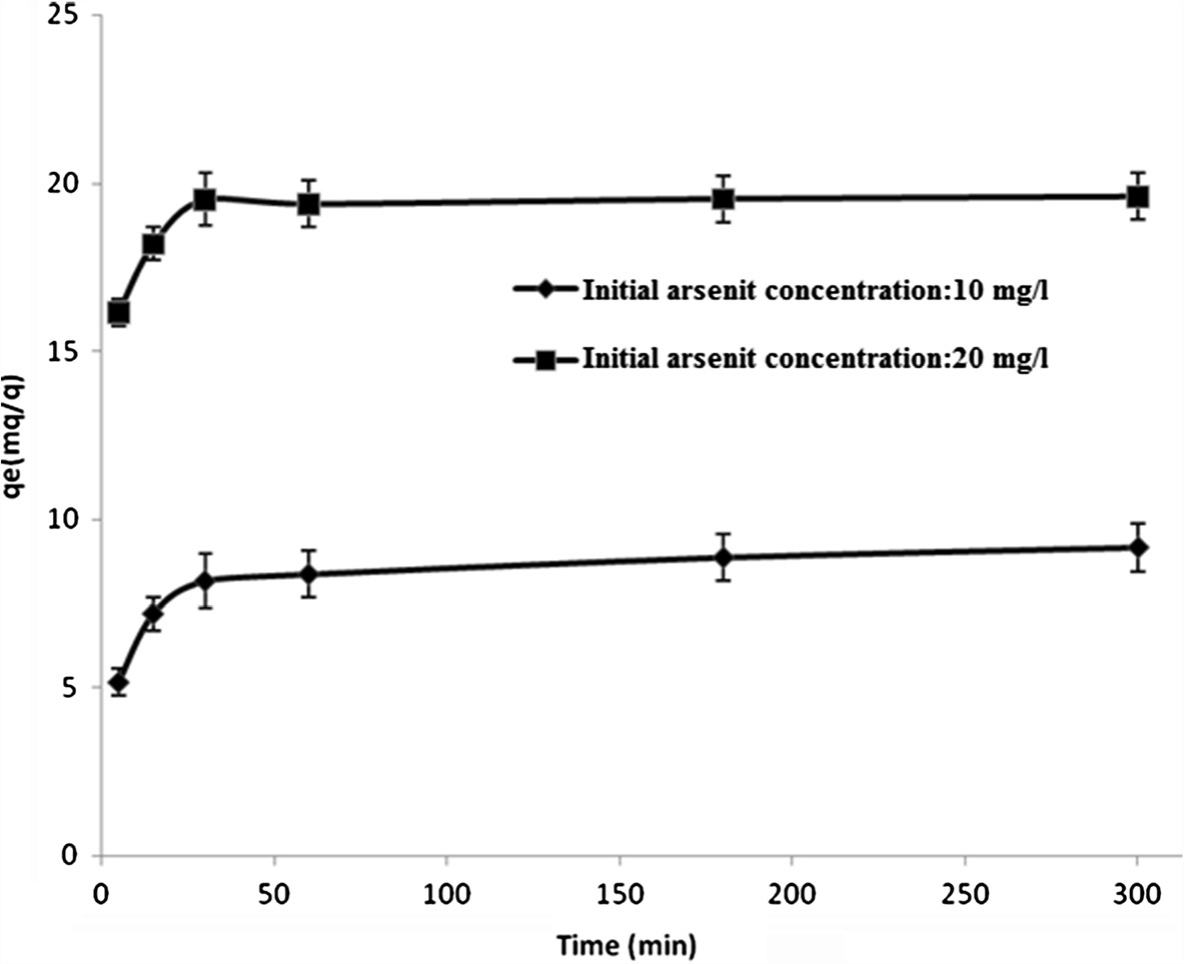
Effect of contact time and initial arsenite concentration on arsenite adsorption (temperature = 25°C, initial arsenite concentration: 10 and 20 mg/L, pH = 7.5, adsorbent dose=1 g/L).

It was observed that in both different initial concentrations of arsenite, removal efficiency increased with time (Figure 7).

The trend of the plots shows that arsenite uptake was rapid in the beginning followed by a slower removal that gradually reached a plateau. Maximum removal of arsenite was achieved within the first 15 min of contact time and equilibrium was reached in 30 min.

There was no important change in arsenite uptake by synthesized nano γ–alumina in the subsequent 6 h.

### Adsorption isotherms

To evaluate the adsorption capacity and for investigating the characteristics of adsorption, the adsorption isotherm is basically important. For this reason, the arsenite adsorption equilibrium data obtained at pH of 7.5 at initial solution concentrations from 5 mg/L to 250 mg/L. When the initial concentration was low, the arsenite concentration in the solution after adsorption was below the detection limit of the spectrophotometer, therefore, the minimum initial concentration of 5 mg/L was selected. Langmuir, Freundlich [28,29] and Dubinin– Radushkevich (D–R) [30] adsorption isotherms were investigated. The Langmuir isotherm model assumes the formation of a monolayer onto the adsorbent surface with a finite number of equations, and the Freundlich isotherm model indicates the heterogeneity of the adsorbent material and these adsorption models are given by the equations given as follows:

(2) $$\frac{C_{e}}{q_{e}}=\frac{C_{e}}{q_{m}}+\frac{1}{q_{m}b}$$

(3) $$q_{e}=K_{f}C^{1/n}$$

For Langmuirisothermmodel, *q*_*e*_ and *q*_*m*_ (mg/g) are the adsorbed and maximum adsorbed amount of arsenite by nano γ–alumina, respectively. C_e_ is the equilibrium concentration (mg/L) of arsenite and b is the Langmuir constant related to energy of sorption. For Freundlich isotherm model, constants n and K_f_ are the Freundlich constants for the system, which were indicators of intensity and adsorption capacity, respectively.

The important characteristics of the Langmuir isotherm are explained by a dimensionless separation factor, RL, which is indicative of the isotherm shape that predicts whether an adsorption system is favorable or unfavorable. RL is defined as [14]:

(4) $${\mathrm{RL}=1/(1+\mathrm{bC}}_{o})$$

The linear form of (D–R) isotherm equation is shown as:

(5) $${\mathrm{Inq}}_{e}{\;=\mathrm{Inq}}_{m}-\beta \epsilon^{2}$$

*q*_*e*_, *q*_*m*_ and *β* are the amount of arsenite adsorbed per unit mass of nano γ–alumina, the theoretical adsorption capacity and the constant of the sorption energy, respectively. Also, *β* is related to the average energy of sorption per mole of the adsorbate as it is transferred to the surface of the solid from infinite distance in the solution, and *ϵ* is Polanyi potential, which is shown as:

(6) $$\epsilon {=\mathrm{RT}\;\mathrm{Ln}(1+1/C}_{e}\;)$$

Where, T and R are the temperature (K) and the gas constant (8.314*10^−3^ kJ/mol.°K. The value of mean sorption energy, E, can be calculated from D–R parameter *β* as follows [31]:

(7) $$E=\frac{1}{\sqrt{-2\beta }}\;$$

For predicting the type of adsorption, the value of E is very useful; if the value is between 1 to 8 kJ/mol, then the adsorption is said to be physical in nature and if it is between 8 to 20 kJ/mol, then the adsorption is said to be chemical in nature [32,33].

**Figure 8:**
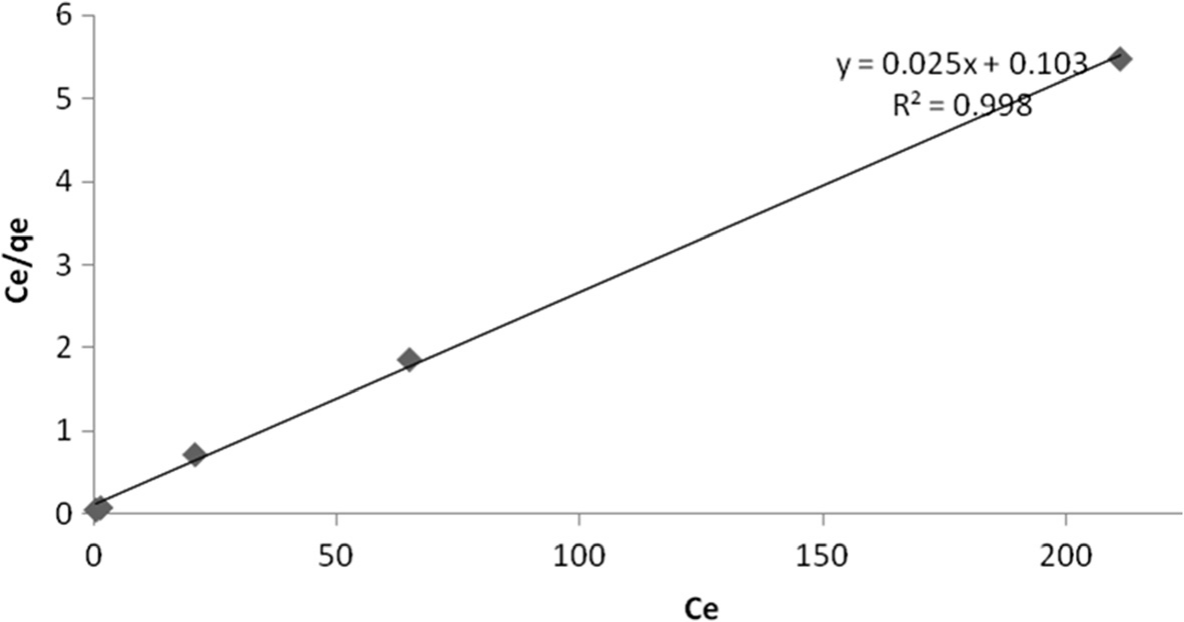
Langmuir isotherm of arsenite sorption on synthesized nano γ–alumina.

Figure 8 illustrates the linear plot of C_e_/q_e_ as a function of C_e_ for the sorption of arsenite on nano γ–alumina, a linear relation with good correlation coefficients (R^2^>0.99) was found. The plots indicate the applicability of Langmuir model in the present study. The values of monolayer capacity (q_m_) and Langmuir constant (b) are given in Table 3. The value of q_m_ calculated by the Langmuir isotherm were all close to experimental values at the given temperature. These facts suggest that arsenite is sorbed in the form of monolayer coverage on the surface of the adsorbent.

**Table 3:** Isotherm constants for arsenite adsorption on synthesized nano γ–alumina

**Langmuir**	**Freundlich**	**D-R**
b=0 .0025	n=4.73	E=7.9 (kJ/mol)
q_m_=40 (mg/g)	K_f_=37.97 (mg/g)	q_m_=36.43 (mg/g)
Rl=0.613		β=0 .008
R^2^=0 .998	R^2^=0.910	R^2^=0. 974

The RL value obtained lie between 0 and 1 confirming that the adsorption isotherm is favorable.

**Figure 9:**
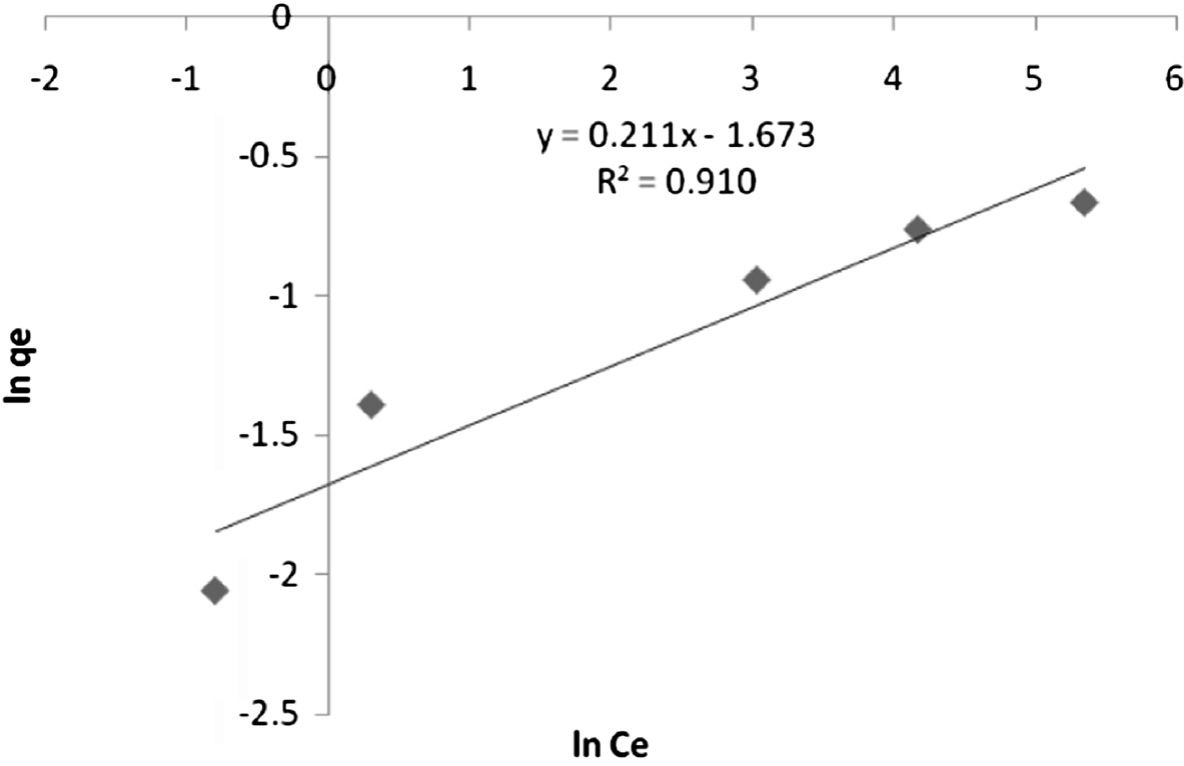
Freundlich isotherm of arsenite sorption on synthesized nano γ–alumina.

For achieving the Freundlich constant, lnq_e_ was plotted against lnC_e_; a straight line with slope 1/n and intercept ln K_F_ was obtained. Figure 9 investigates the linear regression approach for sorption arsenite ions on nano γ–alumina to obtain the model parameters of Freundlich isotherm. From these figure according to the correlation coefficient (R^2^) value, it was demonstrated that the removal of arsenite ions using the nano γ–alumina fairly obeyed the Freundlich isotherm.

The values obtained for the Freundlich, Langmuir and D-R variables for arsenite ions removal are given in Table 3. The n value, higher than 1, shows favorable sorption for arsenite ions using the synthesized nano γ–alumina. The value of the q_m_, that is an indicator of the sorption capacity supports the previous notations that are discussed in the Langmuir isotherm for the sorption capacities of the studied ions removed using the synthesized nano γ–alumina.

**Figure 10:**
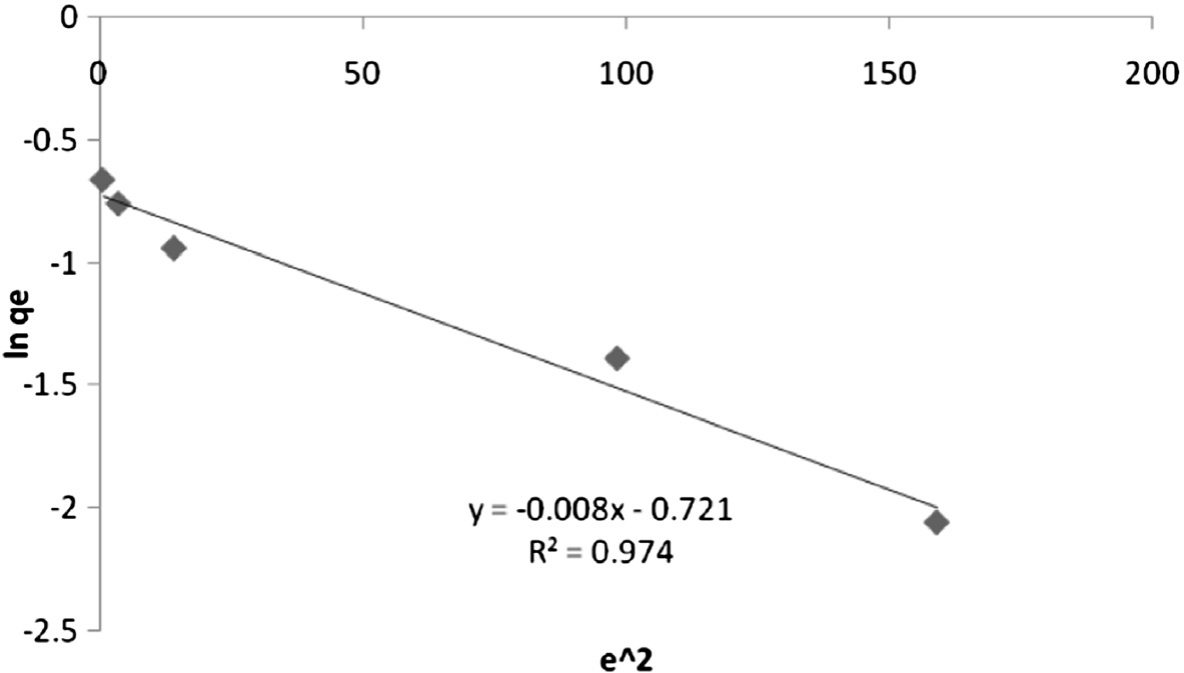
D-R isotherm of arsenite sorption on synthesized nano γ–alumina.

To describe the sorption isotherms of single solute systems, D–R isotherm is commonly used. The D–R isotherm, apart from being analog of Langmuir isotherm, is more general than Langmuir isotherm as it rejects the homogeneous surface or constant adsorption potential [34]. Figure 10 shows the plot of lnq_e_ versus ϵ^2^. The D–R plot yields a straight line with R^2^ value of 0.974, indicating that the D–R model is less fitting to the experimental data as comparable to the Langmuir isotherm model but is better than Freundlich isotherm model.

From the plotted D–R isotherm figure, the model parameters; sorption capacities (q_m_), sorption energy constants (*β*) and the main adsorption energies (E) are listed in Table 3. The value of E was found to be 7.9 kJ mol^−1^ suggesting the physical nature of the adsorption process of arsenite on synthesized nano γ–alumina. So It is important to note that the suitability of all the three isotherm models to the studied sorption systems shows that monolayer sorption (Langmuir and D-R isotherms) of active sites on the surface of the synthesized nano γ–alumina in comparison to heterogeneous energetic distribution (Freundlich isotherm) is more significant [21].

### Kinetic modeling of arsenite removal

The kinetics of arsenite sorption on synthesized nano γ–alumina was analyzed using pseudo-second-order Kinetic model [35,36]. Because for many adsorption processes the Lagergren pseudo first order model is suitable only for the initial 20 to 30 min of interaction and is not suitable for the whole range of contact time [37], in this article only the pseudo-second order equation was evaluated. One of the best advantages of the pseudo-second order equation is that, the amount of arsenite adsorbed can be estimate with small sensitivity for the influence of the random experimental errors [37].At initial arsenite concentration of 10 and 20 mg/L, 97.65 and 94.38% arsenite removal were achieved, respectively in the first 30 minutes at a pH of 7.5. The pseudo-second-order equation can be shown as:

(8) $$\frac{t}{q_{t}}=\frac{1}{K_{2}q_{e}^{2}}+\frac{1}{q_{e}t}$$

Where *K*_2_ (gm/g. min) is the rate constant of pseudo-second order adsorption and often depends on the applied operating conditions, such as, pH of solution, initial metal concentration, temperature and agitation rate, etc. [38,39]. From the slope and intercept of a plot of t/*q*_*t*_ against t, the initial adsorption rate, h, adsorption capacity (*q*_*e*_) and the pseudo-second order rate coefficient (*K*_2_) was determined and are shown in Table 4.

**Table 4:** Pseudo-second-order rate constants

**Arsenite conc. (mg/L)**	**q**_**e**_**(exp.) (mg/g)**	**q**_**e**_**(cal.) (mg/g)**	**K**_**2**_**(g/g.min)**	**R**^**2**^	**h**
10	9.15	9.256	0.02	0.99	1.85
20	19.61	20	0.050	1	22.7

The initial adsorption rate, h, of a second order process as t→0 can be shown as:

(9) $$h=K_{2}q_{e}^{2}$$

**Figure 11:**
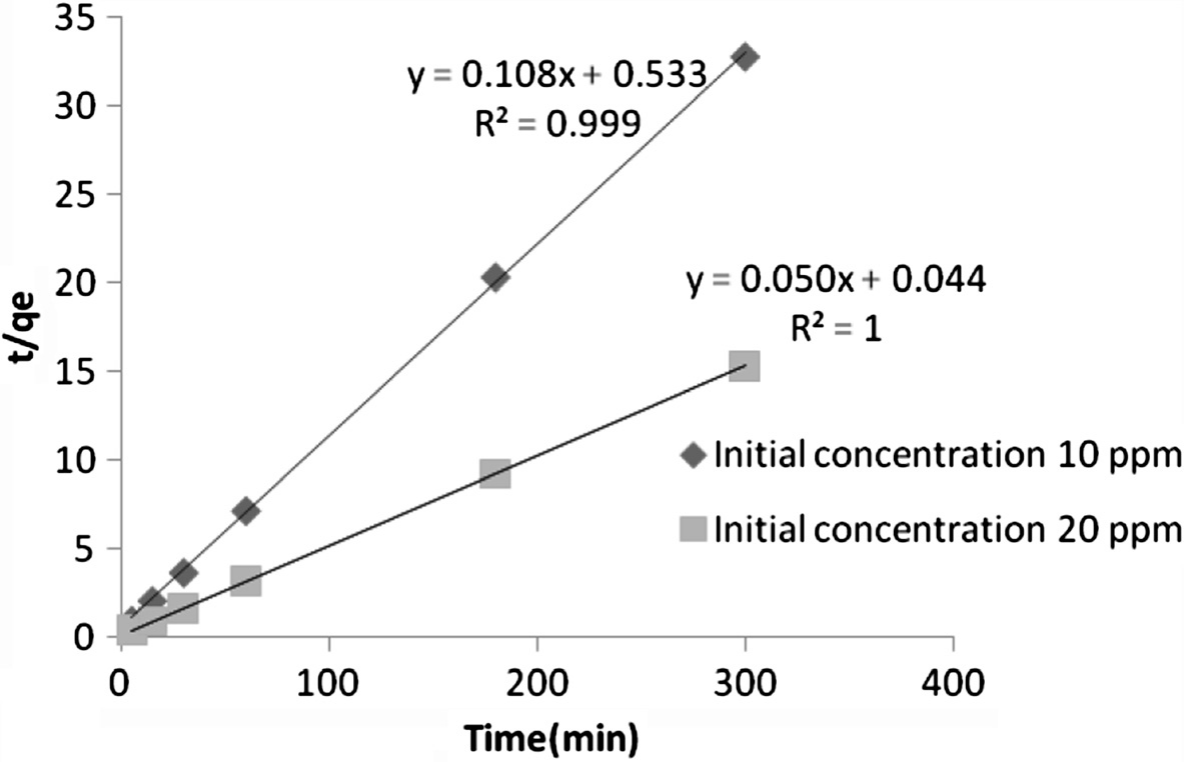
Pseudo-second-order kinetic plots of sorption of arsenite on synthesized nano γ–alumina.

The values obtained by pseudo-second-order model (Figure 11) were found to be in good agreement with experimental data and can be used to favorably explain the arsenite sorption on synthesized nano γ–alumina and the rate constant of sorption process seems to be controlled by the physical sorption process.

### Comparison with other adsorbents

It has been reported that the maximum arsenite adsorption capacity of Iron hydroxide coated alumina, activated alumina grains and manganese oxide-coated alumina are 9, 3.48 and 42.48 mg/g, respectively [9,40,41], which is listed in Table 5.

**Table 5:** Comparison of maximum arsenite adsorption capacities for different adsorbents

**Adsorbent**	**Conc. range (mg/L)**	**q**_**m **_**(mg/g)**	**pH**	**Surface area (m**^**2**^**/g)**	**Ref.**
Iron hydroxide coated alumina	7.5- 135	9	6.6	---	[40]
Activated alumina	1	0.18	7.6	370	[35]
Activated alumina grains	0.79-4.9	3.48	7	117	[9]
Manganese oxide- coated alumina	2-300	42.48	4-7.5	194.09	[41]
Nano γ–alumina	10-250	40	7.5	201.53	Present study

In comparison, it is clear that maximum arsenite adsorption capacity of synthesized nano γ–alumina is bigger than Iron hydroxide coated alumina and activated alumina grains, and it is a little smaller than manganese oxide-coated alumina adsorbent.

Although the adsorption capacity of arsenite onto synthesized nano γ–alumina adsorbent is lower than that of manganese oxide-coated alumina, but the main advantages of synthesized nano γ–alumina adsorbent are the availability of the materials, the substantially low cost, and its economic feasibility. This suggests that the adsorption property of synthesized nano γ–alumina gives the material great potential for applications in arsenite removal from aqueous solutions.

## Conclusion

A simple method for producing nano γ–alumina from impure kaolin with spherical shape, with a particle size distribution ranging from 22 to 36 nm with a relatively high surface area (201.53 m^2^/g) and its application for arsenite removal without pre-oxidation of arsenite is described in this study.FT-IR analyses reveal that the arsenite cannot be adsorbed onto synthesized nano γ–alumina chemically. Besides, the results show that arsenite ions are physically adsorbed on the surface of synthesized nano γ–alumina. About 97.65% of arsenite removal is achieved within 30 min from the samples containing initial arsenite concentration up to 10 mg/L at pH =7.5. It is also revealed that the experimental results of adsorption isotherms are well fitted with the Langmuir and D-R models and the maximum adsorption capacity was 40 mg/g. The adsorption rate of arsenite is fast and equilibrium time is around 15 min. The pseudo-second-order model is suitable for all initial arsenite concentrations (10 and 20 mg/L), suggesting that the adsorption of arsenite onto synthesized nano γ–alumina follows pseudo-second-order kinetics.

## Competing interests

The authors declare that they have no competing interests.

## Authors‘ contributions

All authors read and approved the final manuscript.
